# Recent Advances in Optoelectronic Synaptic Devices for Neuromorphic Computing

**DOI:** 10.3390/biomimetics10090584

**Published:** 2025-09-03

**Authors:** Heeseong Jang, Seohyeon Ju, Seeun Lee, Jaewoo Choi, Ungbin Byun, Kyeongjun Min, Maria Rasheed, Sungjun Kim

**Affiliations:** 1 Division of Electronics and Electrical Engineering, Dongguk University, Seoul 04620, Republic of Korea; 2 Department of Advanced Battery Convergence Engineering, Dongguk University, Seoul 04620, Republic of Korea

**Keywords:** optoelectronic, trap, dimensional materials, synaptic, neuromorphic, application

## Abstract

We explore recent advancements in optoelectronic synaptic devices across four key aspects: mechanisms, materials, synaptic properties, and applications. First, we discuss fundamental working principles, including oxygen vacancy ionization, defect trapping, and heterojunction-based charge modulation, which contribute to synaptic plasticity. Next, we examine the role of 0D, 1D, and 2D materials in optimizing device performance, focusing on their unique electronic, optical, and mechanical properties. We then analyze synaptic properties such as excitatory post-synaptic current (EPSC), visual adaptation, transition from short-term to long-term plasticity (STP to LTP), nociceptor-inspired responses, and associative learning mechanisms. Finally, we highlight real-world applications, including artificial vision systems, reservoir computing for temporal data processing, adaptive neuromorphic computing for exoplanet detection, and colored image recognition. By consolidating recent developments, this paper provides insights into the potential of optoelectronic synaptic devices for next-generation computing architectures, bridging the gap between optics and neuromorphic engineering.

## 1. Introduction

Over the past several decades, the rapid advancement of information technology has transformed nearly every aspect of modern life. However, as Moore’s law approaches its physical limits, conventional computing architectures are increasingly struggling to meet the demands of modern data-intensive applications, including artificial intelligence (AI), big data processing, and real-time analytics [1,2,3]. The fundamental limitation of the von Neumann architecture arises from the physical separation between processing and memory units, which results in high power consumption and inefficiencies in data transfer. This constraint is commonly referred to as the von Neumann bottleneck [4]. To overcome these limitations, researchers have been actively exploring alternative computing paradigms that break away from traditional architectures [5,6]. One promising approach is brain-inspired neuromorphic computing, which aims to emulate the human brain’s exceptional capability for high-speed parallel processing while maintaining low energy consumption [7,8,9]. Unlike conventional architectures, where computation and memory storage are separate, the human brain consists of approximately 10^12^ neurons interconnected by 10^15^ synapses, allowing for efficient integration of storage and computation. This structure allows biological brains to dynamically process complex information, adapt to new inputs, and perform cognitive tasks such as learning, reasoning, and perception with remarkable energy efficiency [10,11]. To replicate such neuromorphic functionalities, significant efforts have been devoted to developing artificial synaptic devices based on emerging memory technologies. Among them, memristors have attracted considerable attention due to their ability to mimic synaptic plasticity and facilitate in-memory computing [12,13,14]. However, the majority of memristors are purely electronic devices that rely on electrical input and output, which presents several fundamental limitations [15,16,17]. These electronic synapses suffer from narrow bandwidth, interconnect-related resistance–capacitance (RC) delay, and power dissipation, all of which degrade performance and scalability. RC delay, in particular, becomes increasingly problematic as device density increases, limiting the speed at which information can be transmitted across a neuromorphic network [18,19]. Additionally, electronic-based memristors are sensitive to thermal effects, which further increase power loss and affect long-term stability [20,21].

To address these challenges, optoelectronic and all-optical memristors have emerged as promising alternatives, utilizing light as a medium for computation and memory operations [22,23,24,25,26]. Optical memristors utilize light–matter interactions in materials such as semiconductors, oxides, graphene, perovskites, and black phosphorus to achieve synaptic functions [27,28,29,30]. These devices offer significant advantages over conventional electronic counterparts, including higher bandwidth, ultrafast response times, and reduced energy dissipation [31,32]. Optoelectronic neuromorphic computing further integrates visual inputs as pre-synaptic stimuli, allowing for dynamic synaptic weight modulation based on light-induced responses [33,34]. In this context, photonic synapses have emerged as a crucial category of devices, where light pulses act as the primary stimulus, facilitating efficient signal processing and neural transmission at the device level [35,36,37]. Moreover, recent advancements in bio-visual perception systems (BVPSs) and electroluminescent synaptic devices have further expanded the scope of optoelectronic computing [38,39]. These technologies utilize optical signals both as input stimuli and modulators, allowing for more energy-efficient and scalable neuromorphic architectures [40,41]. As research continues to push the boundaries of optoelectronic neuromorphic computing, optical memristors hold immense potential for revolutionizing artificial intelligence, vision systems, and next-generation computing architectures by overcoming the limitations of traditional semiconductor-based technologies [42,43,44].

**Figure 1:**
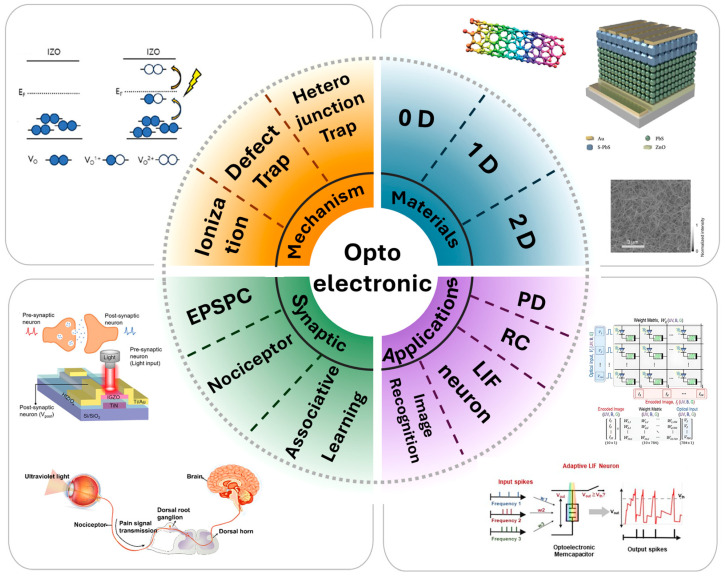
Schematic representation of this review [45,46,47,48,49,50,51,52], which are described in detail later in the text.

In this review, we explore recent advancements in optoelectronic synaptic devices, categorized into four key aspects: mechanism, materials, synaptic properties, and applications. First, we discuss the fundamental mechanisms underlying optoelectronic synaptic behavior, including oxygen vacancy ionization/deionization, defect-mediated charge trapping, and heterojunction-based carrier modulation. Next, we examine the role of material dimensionality (0D, 1D, 2D) in governing the electronic, optical, and mechanical properties of optoelectronic synaptic devices. The careful selection of materials is crucial for optimizing charge transport, light absorption, and overall device performance. We then analyze the synaptic properties of these devices, focusing on their ability to mimic biological vision systems. Key topics include excitatory post-synaptic current (EPSC), visual adaptation, the transition from short-term to long-term plasticity, nociceptor-inspired responses, and associative learning mechanisms. Finally, we explore applications of optoelectronic synaptic devices, including artificial vision systems, reservoir computing (RC) for temporal data processing, adaptive neuromorphic computing for exoplanet detection, and colored image recognition [44]. For clarity, Table 1 summarized recently reported optoelectronic synaptic devices for comparison, and Table 2 lists all acronyms used in this work. Schematic representation of this review is shown in Figure 1. These advancements highlight the potential of optoelectronic neuromorphic devices for next-generation computing architectures.

**Table 1:** Recently reported optoelectronics devices for comparison.

Type	Device Structure	Availability of Stimuli	Wavelength (nm)	Synaptic Functions	Application	Energy Consumption	Ref.
ReRAM	Au/GO-TiO_2_/ITO	All optical	380 nm	STP, PPF	Image sensing, sensorimotor system	4.5 mW/cm^2^	[53]
ReRAM	ITO/ZTO/In_2_O_3_	Electrical/Optical	405 nm	STM to LTM, Relearning	Mimicking human eye object tracking	6.4 mW/cm^2^	[28]
FET	Graphene/MoS_2_/ SiO_2_/Au/SiNx/Si	All optical	395, 660 nm	STM to LTM, PPF	Polarization imaging system	3.0 mW/cm^2^	[54]
Phototransistor	MoS_2_/ZnO/Cr/Au	All optical	375, 490, 525 nm	PPF, EPSC, Nociceptor, Pavlov, Logic gate	-	2.55 × 10^−9^ J	[55]
ReRAM	PET/ITO/C_60_@GO/ITO/Au	All optical	395 nm	PPF, EPSC	Memory-dependent dynamic vision recognition	8.0 mW/cm^2^	[56]
Three-terminal transistor	WSe_2_/SnSe_2_/Cr/Au/SiO_2_/Si	All optical	400, 500, 600 nm	PPF, STM to LTM	PD MNIST	47.5 pJ	[57]
ReRAM	Au/CsCu_2_I_3_/PEDOT:PSS/ITO/Glass	All optical	445 nm	PPF, EPSC	RC, elderly fall detection	18 nJ	[58]
Three-terminal transistor	Au/Mo_1−x_W_x_S_2_/SiO_2_/Si	All optical	532 nm	PPF, Relearning	ANN for image recognition	27.3 mW/cm^2^	[59]
ReRAM	ITO/NiO/IGZO/Pt	All optical	470 nm	PPF, EPSC	RC, spoken-digit recognition	20.3 mW/cm^2^	[60]
ReRAM	Pd/ZnO/SnSe/ITO	All optical	405~1550 nm	Relearning, STM to LTM, Logic gate	-	15.0 mW/cm^2^	[61]
Three terminal transistor	Al_2_O_3_/MoS_2_/PTCDA/Si/SiO_2_	Electrical/Optical	532 nm	IPSC, EPSC, PPD, PPF, SRDP	dynamic filtering	10 pJ	[62]
Two terminal transistor	Al/Si Nc/ITO	Electrical/Optical	375~1870 nm	EPSC, PPF, STDP	-	0.7 pJ	[63]
Two terminal transistor	ITO/ZnO QD/CdSe QD/ZnO QD/Al	Electrical/Optical	365 nm	EPSC, PPF	Image color perception, RC	-	[64]
ReRAM	ITO/ZnO/HfO_2_/ITO	Electrical/Optical	405 nm	MLC, Potentiation, PPF, STDP	ANN for pattern recognition	-	[65]
Three terminal transistor	Au/Cs_2_AgBiBr_6_/Si/SiO_2_	All Optical	532, 660 nm	PPF, PPD potentiation, depression	Digit recognition	-	[66]
ReRAM	Ag/Ga_2_O_3_/MoS_2_/ITO	Electrical/Optical	365 nm	EPSC, PPF, STM to LTM	Perception-memory system	180 pJ	[67]
Two terminal transistor	ZnO/Ag-nanowires/PET	All Optical	365 nm	EPSC, PPF, PPD, STDP, Pavlov	-	4 μJ	[68]
Three terminal transistor	Au/C_3_N_4_/PMMA/NT-CN/Si/SiO_2_	All Optical	365, 460, 520, 625 nm	EPSC, PPF	UV-transmittance modulator	18.06 fJ	[69]
Three terminal transistor	ZnO/PVSK/InO_x_/Li-AlO_x_	Electrical/Optical	465, 525, 625 nm	EPSC, PPF,	ANN for image recognition	1.38 nJ	[70]
Three terminal transistor	MoS_2_/CdSe/ZnS/SiO_2_	All Optical	460 nm	EPSC, PPF, PPD, potentiation, depression		62 μW/cm^2^	[71]

**Table 2:** Acronyms and definitions used in this work.

Acronym	Full Name	Description
EPSC	Excitatory Postsynaptic Current	Postsynaptic current generated in response to excitatory input, used to quantify synaptic strength.
PPC	Persistent Photoconductivity	Long-lived increase in electrical conductivity after illumination is turned off.
STP	Short-Term Plasticity	Transient change in synaptic strength lasting from milliseconds to minutes.
LTP	Long-Term Plasticity	Persistent change in synaptic strength lasts from hours to days.
LTP	Long-Term Potentiation	Specific types of long-term plasticity characterized by increased synaptic strength.
LTD	Long-Term Depression	Activity-dependent reduction in synaptic strength.
STM	Short-Term Memory	Information storage lasts a short period, typically seconds to minutes.
LTM	Long-Term Memory	Information storage lasts from hours to years.
MeC	Memory Current	Current level representing stored conductance state in a synaptic device.
MeD	Memory Degree	Quantitative measure of memory retention in a synaptic device.
ReC	Response Current	Current generated in immediate response to a stimulus, used to assess device sensitivity.

## 2. Mechanism

### 2.1. Ionization and Deionization of Oxygen Vacancies

Oxide semiconductors are widely utilized in both all-electronic and optoelectronic synaptic devices due to their inherent persistent photoconductivity (PPC) [72,73]. The PPC effect enables these materials to exhibit synaptic plasticity under optical stimulation, making them promising candidates for neuromorphic computing applications. A fundamental aspect of PPC is the ionization and deionization of oxygen vacancies (Vo), which significantly influence the conductivity of oxide semiconductors. Oxygen vacancies, which are intrinsic defects in the crystal lattice of oxide semiconductors, can exist in multiple charge states, including neutral and positively charged configurations [74,75,76]. Their ionization and deionization behavior is influenced by external stimuli such as light or electrical bias, as well as interactions with neighboring atoms. Under optical excitation, oxygen vacancies tend to become positively charged due to electron excitation. Once the light source is removed, a gradual deionization process occurs, during which electrons are recaptured by the vacancies, returning them to their neutral state. This transition between ionized and neutral states directly affects the material’s electronic properties, leading to long-term synaptic plasticity. Despite extensive research, the precise mechanisms governing the generation and recombination of photogenerated carriers in oxide semiconductors remain an open question. However, a widely accepted view suggests that light stimulation ionizes oxygen vacancies, resulting in an increase in conductivity, followed by a slow deionization process after the cessation of illumination. This delayed recombination of carriers contributes to the extended retention of photocurrent, mimicking the gradual decay of synaptic responses observed in biological systems.

**Figure 2:**
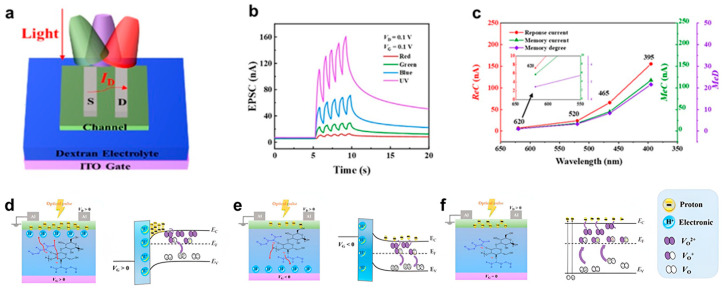
(**a**) 3D diagram of the dextran-OST and the principle of optoelectronic regulation; (**b**) EPSC under different light wavelengths; (**c**) The ReC, MeC, and MeD of the dextran-OSTs. The ReC & MeC & ReD at different light wavelengths. The structure and energy band diagram of dextran-OSTs; (**d**) VG > 0 V. (**e**) VG < 0 V. (**f**) VG = 0 V [77].

Recently, Huang, Bo et al. have investigated synaptic responses to optical stimuli at different wavelengths to investigate the optical plasticity of IGZO-based devices (Figure 2a) [77]. As shown in Figure 2b, exposure to five consecutive ultraviolet (UV) light pulses (395 nm) induced the highest EPSC, whereas in visible light stimulation (blue: 465 nm, green: 520 nm, red: 620 nm), EPSC levels were lowered (UV > blue> green > red). This response can be attributed to the change in the probability of photoexcitation with wavelength. Due to the high density of oxygen-vacancy associated defect states (10^19^–10^20^ cm^−3^) near the valence band maximum (VBM), defect-mediated photoexcitation is possible due to the ease of sub-gap light absorption. However, as the incident light wavelength increases, the electron excitation probability decreases, resulting in a decrease in the EPSC response. Specifically, red and green light with photon energy below 2.3 eV exhibits lower photoexcitation efficiency compared to blue and ultraviolet light with higher photon energy that can directly excite electrons into the conduction band of IGZO. The memory characteristics of IGZO-based synaptic devices were further analyzed using response current (ReC), memory current (MeC), and memory degree (MeD) measurements (Figure 2c). The results indicate that shorter-wavelength light induces stronger memory effects, consistent with the higher probability of defect-assisted excitation at higher photon energies. Additionally, to simulate real-world visual adaptation, white light (30 μW/cm^2^) was applied to the device, demonstrating that synaptic responses can be dynamically modulated by gate voltage (V_G_). As depicted in Figure 2d–f, when a positive gate bias (V_G_ > 0) is applied, protons (H^+^) accumulate at the interface of the channel and dielectric layer, facilitating the participation of oxygen vacancies in charge transport and enhancing the EPSC. Conversely, when a negative gate bias (V_G_ < 0) is applied, partial recombination of oxygen vacancies occurs, leading to a reduction in EPSC. Notably, even at V_G_ = 0, the device retains residual EPSC, which can be attributed to the strong PPC effect of IGZO. This phenomenon suggests that a substantial fraction of ionized oxygen vacancies remains active in conduction pathways, sustaining synaptic behavior even in the absence of external bias.

These findings highlight the crucial role of oxygen vacancy ionization in the optoelectronic response of IGZO-based synaptic devices. The ability to control optical synaptic behavior via wavelength-dependent excitation and electrostatic modulation provides a promising avenue for developing energy-efficient neuromorphic vision systems.

### 2.2. Defect Traps

Trap states in semiconductor-based optoelectronic devices play a crucial role in regulating carrier transport by capturing and releasing charge carriers such as electrons and holes [78,79,80]. These traps, often associated with defects like dangling bonds or structural distortions, contribute to synaptic plasticity by modulating the device’s conductivity. Upon optical stimulation, electron-hole pairs are generated, and while some electrons recombine immediately, others become trapped in defect states at the semiconductor interface. These trapped carriers can later be thermally released into the conduction band, influencing the decay characteristics of the photocurrent. Repeated optical stimulation before complete carrier detrapping can lead to the occupation of lower-energy trap states, extending decay time and enabling long-term memory characteristics in artificial synapses. This trapping/detrapping mechanism is essential for mimicking biological synaptic behaviors and improving the retention of optically encoded information in neuromorphic devices.

**Figure 3:**
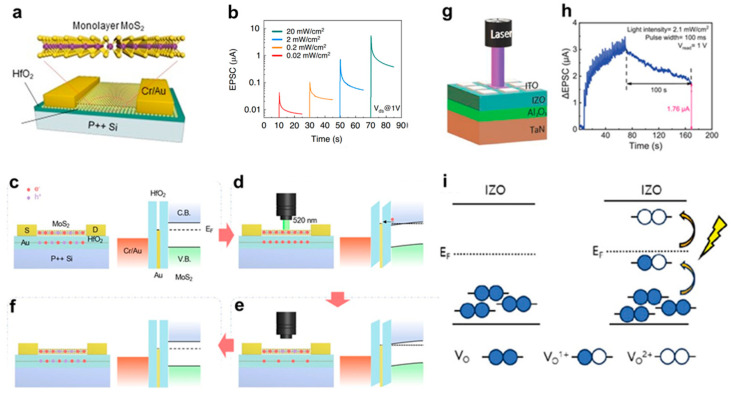
(**a**) Three-dimensional schematic illustration of the MoS_2_ floating-gate artificial synaptic device structure; (**b**) Variation in EPSC under different optical power densities. The band energy of the device is modulated by laser illumination; (**c**–**f**) Sequential illustration of the device’s operational states, showing the initial state before laser exposure, response under 520 nm laser irradiation, the state immediately after laser removal, and the gradual recovery to the original state. Arrows in (**c**–**f**) denote the state-transition sequence [81]; (**g**) Schematic representation of optical measurements using with a pulse of UV light; (**h**) EPSC response observed after applying 30 pulses at 100 ms intervals; (**i**) Illustration of the oxygen defect modulation in IZO induced by light exposure [45].

Zhang et al. demonstrated a highly uniform artificial visual neural network using a wafer-scale single-layer MoS_2_ floating gate field-effect transistor (FGFET) array (28 × 28 devices, 0.7 × 0.7 cm^2^), as shown in Figure 3a [81]. In this system, the MoS_2_ channel functions analogously to the pre-synaptic membrane, responding to light stimuli, while the intra-channel current between the source and drain electrodes corresponds to EPSC. When a light spike is applied, photogenerated carriers increase the channel conductivity, and some of these carriers are captured by tunneling into the floating gate layer. Upon removal of the light stimulus, the captured charges are gradually released, allowing the current to return to its initial state. This charge modulation process, facilitated by Au NPs in the floating gate, mimics synaptic weight modulation and neurotransmitter regulation in biological synapses. Figure 3b illustrates the dependence of EPSC on optical power density, where the current response increases significantly from approximately 0.03 μA to 5 μA as the power density varies from 0.02 to 20 mW/cm^2^. Figure 3c–f detail the working mechanism of the optical synaptic device. Initially, the device remains in equilibrium, maintaining a stable Fermi level in the MoS_2_ channel. Upon light exposure, electron-hole pairs form at the MoS_2_ surface, leading to electron accumulation and a downward shift in the energy band. Simultaneously, some electrons undergo Fowler-Nordheim tunneling into the Au NPs floating gate layer. After the light stimulus is removed, while the initial conductivity enhancement diminishes, the trapped electrons in the floating gate do not immediately return, causing a gradual depletion of free carriers in the MoS_2_ channel. Over time, these trapped charges are slowly released, resulting in a progressive recovery of PSC. However, due to the trapping effect within the floating gate, this recovery process is delayed. Eventually, as all trapped electrons are fully released, the device returns to its original state. This sequence of events effectively replicates the pre-synaptic and post-synaptic processes observed in biological synapses, with electrical signals sequentially propagating through the Au NPs floating gate layer, the HfO_2_ tunnel layer, and the MoS_2_ channel.

Park, Hyoeun et al. demonstrated the mimicry of synaptic properties in ITO/IZO/Al_2_O_3_/TaN devices through both electrical and optical stimuli, highlighting the coexistence of short-term memory (STM) and long-term memory (LTM) (Figure 3g) [45]. As shown in Figure 3h, repeated optical stimulation (100 ms pulse width, 2.1 mW/cm^2^ intensity, 30 pulses) led to prolonged synaptic strengthening, attributed to charge trapping mechanisms. The underlying working principle is illustrated in Figure 3i, where IZO, an amorphous oxide semiconductor, undergoes ionization of oxygen vacancies (Vo → Vo^1+^, Vo^2+^) upon exposure to light. When photons with energy exceeding the bandgap of IZO are absorbed, the material transitions from its neutral ground state to a metastable ionized state, increasing conductivity. Upon removal of light, the system gradually returns to equilibrium via a thermally activated process, resulting in a slow decay of current. This delayed recovery, governed by the trapping and de-trapping of carriers, plays a key role in the synaptic plasticity of the device.

### 2.3. Heterojunction Traps

In recent years, heterostructures have gained significant attention as a means to modulate optoelectronic properties in materials. The precise design of interfacial band alignments and the strategic assembly of heterojunctions enable control over photogenerated charge carriers, including their selective trapping in specific layers. This approach is particularly beneficial in preventing charge saturation in read circuits, which is a critical concern in narrow-bandgap semiconductors. Moreover, it enhances EPSC and suppresses carrier recombination, leading to a gradual decay in photocurrent, which is essential for achieving synaptic plasticity.

**Figure 4:**
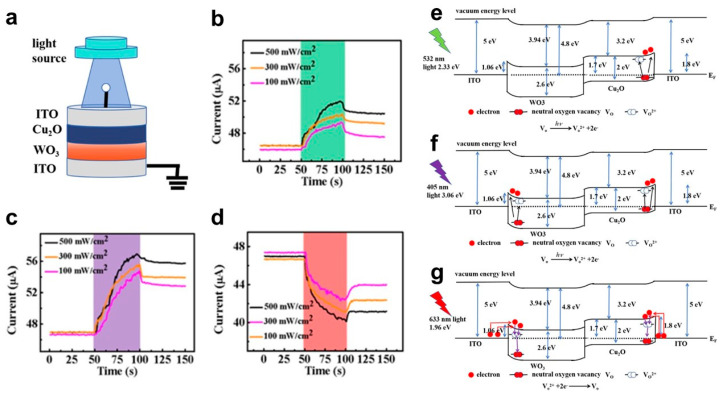
(**a**) Schematic diagram of the device under light illumination; Current response under the application of (**b**) 405, (**c**) 532, and (**d**) 632 nm wavelength light irradiation; Light-tunable mechanism of (**e**) 532 nm (green light), (**f**) 405 nm (purple light), and (**g**) 633 nm (red light), respectively [83].

Shrivastava, Saransh et al. introduced a photonic memory device utilizing a Ag/ITO/Ga_2_O_3_/ZnO/ITO/Glass structure, which functions as a resistive random-access memory (RRAM) device with synaptic properties [82]. Wu Facai et al. presented the demonstration of all-optically controlled resistive switching behavior in an optoelectronic memristor composed of a CMOS-compatible ITO/Cu_2_O/WO_3_/ITO structure (Figure 4a) [83]. As depicted in Figure 4b,c, the photocurrent exhibits a gradual increase under 532 nm light illumination, rising from 46 μA to 49, 50, and 52 μA as the light intensity increases to 100, 300, and 500 mW/cm^2^, respectively. In contrast, the device’s response to 633 nm red light follows an unexpected trend. As shown in Figure 4d, when exposed to red light at intensities of 100, 300, and 500 mW/cm^2^, the current steadily decreases over time from an initial value of 47 μA. After 50 s of illumination and subsequent light-off, the current recovers and stabilizes at lower values of 44, 42, and 41 μA, respectively. This behavior is in stark contrast to the current enhancement observed under 405 and 532 nm light excitation. The reason for this phenomenon is that the switching behavior of the device is related to the generation and extinction of light-induced VO^2+^. As illustrated in Figure 4e, when exposed to green light (~2.33 eV), the neutral oxygen vacancies in Cu_2_O, which has an energy band gap of 2 eV, absorbs photon energy, become excited to the conduction band, and subsequently transition into VO^2+^. With extended illumination, the accumulation of VO^2+^ within the Cu_2_O layer promotes the formation of conductive filaments (CFs), thereby enhancing the optical conductance. Under purple light (~3.06 eV) (Figure 4f), which possesses higher photon energy than the band gaps of both Cu_2_O and WO_3_ (~2.6 eV), VO^2+^ formation occurs in both layers, further influencing the conduction mechanism. However, when the device is exposed to red light (~1.96 eV) (Figure 4g), the photon energy is insufficient to excite electrons across the band gaps of Cu_2_O and WO_3_, preventing direct conduction band excitation. Nevertheless, since the red light energy surpasses the Schottky barrier height at the ITO/WO_3_ and Cu_2_O/ITO interfaces, electrons from ITO can surmount the barrier and enter the conduction band. A portion of these electrons is subsequently captured by VO^2+^, reverting them to neutral oxygen vacancies, ultimately leading to a reduction in optical conductance.

## 3. Materials

In optoelectronic devices, the dimensionality of materials plays a crucial role in determining their fundamental properties and performance. Materials can be categorized in zero-dimensional (0D), one-dimensional (1D), and two-dimensional (2D) materials. By the choice of materials electronic, optical, and mechanical characteristics can change significantly, such as charge transport, light absorption, and the overall efficiency of the devices. Therefore, choice of material dimensionality is essential for optimizing the performance of optoelectronic applications. A summary of representative 0D, 1D, and 2D optoelectronic synaptic devices and their key performance metrics is provided in Table 3.

### 3.1. 0D Materials

0D materials, such as quantum dots and nanoclusters, play a pivotal role in optoelectronic devices due to their unique quantum mechanical properties. It is confined to nanoscale dimensions, exhibit discrete energy levels that result from quantum confinement, leading to size-tunable optical and electronic behaviors [93,94]. By adjusting the material’s size, the energy band gap can be controlled, allowing for precise modulation of light absorption and emission, making them ideal for applications like LEDs [95,96,97], solar cells [98,99], and photodetectors [100,101]. Additionally, these materials have a high surface-to-volume ratio, enhancing their optical and electronic responsiveness. This characteristic makes 0D materials highly efficient in light absorption and emission processes, crucial for applications that require sensitivity to external stimuli. Their outstanding optical properties, such as high quantum efficiency and strong photoluminescence, further highlight their suitability for advanced optoelectronic devices. In conclusion, the unique properties of 0D materials, including tunable optical characteristics and high surface reactivity, make them essential for the development of efficient optoelectronic devices.

One of recent studies, Balamur et al. [102] developed a retina-inspired optoelectronic synapse by integrating quantum dots (QDs) into a photonic synaptic array, capable of photon detection, synaptic potentiation/depression, and neural-like processing under visible to UV light stimuli. These devices demonstrated fast response, high retention, and reliable synaptic weight adjustment under pulsed optical inputs.

**Figure 5:**
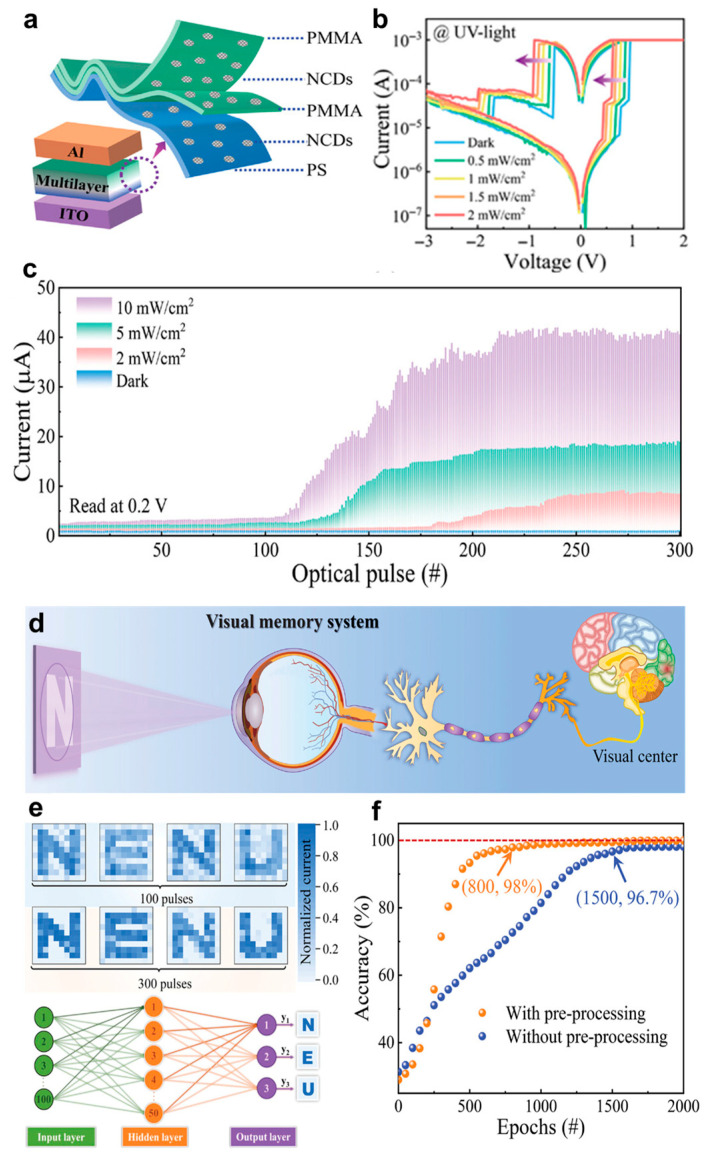
(**a**) Schematic illustration of the carbon-dot-based optoelectronic memristor structure; (**b**) Typical current–voltage curves of the device under UV illumination; (**c**) Analog resistive switching characteristics under optical pulses of varying intensities (#: pulse number); (**d**) Schematic diagrams of visual information processing inspired by the human visual system; (**e**) Example of letter image recognition before and after preprocessing, alongside the artificial neural network structure; (**f**) Improvement in recognition accuracy with preprocessing compared to raw input data (#: Epoch number) [103].

In addition, Xu et al. [103] developed an optoelectronic memristor by incorporating carbon dots (CDs), one of the 0D materials. The device was fabricated by embedding nitrogen-doped CDs within polymethylmethacrylate (PMMA) and polystyrene (PS) layers, forming a multi-stacked structure capable of both digital and analog resistive switching. As shown in Figure 5a, the CDs serve as photoactive charge-trapping centers, and their trapping/detrapping behavior can be modulated by UV illumination. The resulting light-dependent electrical response is evident from the current voltage characteristics (Figure 5b) and the gradual conductance modulation under optical pulses of varying intensities (Figure 5c). Beyond fundamental synaptic properties, the device can be configured into arrays that emulate visual information processing, as demonstrated in Figure 5d. These arrays show image sensing, storage, and contrast enhancement functions inspired by the human visual system. Furthermore, preprocessing of input images using the device significantly improves the accuracy and training efficiency of artificial neural networks for pattern recognition tasks. (Figure 5e,f). These results highlight the versatility and neuromorphic potential of 0D materials such as CDs, which combine tunable optoelectronic characteristics with system-level functions, positioning them as promising building blocks for next-generation neuromorphic optoelectronic applications.

### 3.2. One-Dimensional Materials

Dimensional reduction of 2D materials can result in the formation of 1D nanostructures, such as nanowires (NWs) [104,105], nanotubes (NTs) [47,106], and nanoribbons (NRs) [107], which possess unique properties due to their reduced dimensionality. The dimensions, surface characteristics, and edge atomic arrangements of these 1D materials are essential in determining their electronic behaviors and catalytic activities. For example, materials like III-V semiconductor nanowires and organic nanowires can vary in diameter from just a few nanometers to hundreds of nanometers, while their lengths can span from micrometers to even tens of micrometers [108,109]. These 1D structures offer several advantages, including a high surface-to-volume ratio, excellent charge carrier mobility, antenna-like geometry, and subwavelength size effects, which result in enhanced photoelectric conversion efficiency and tunable light absorption properties [110,111].

**Figure 6:**
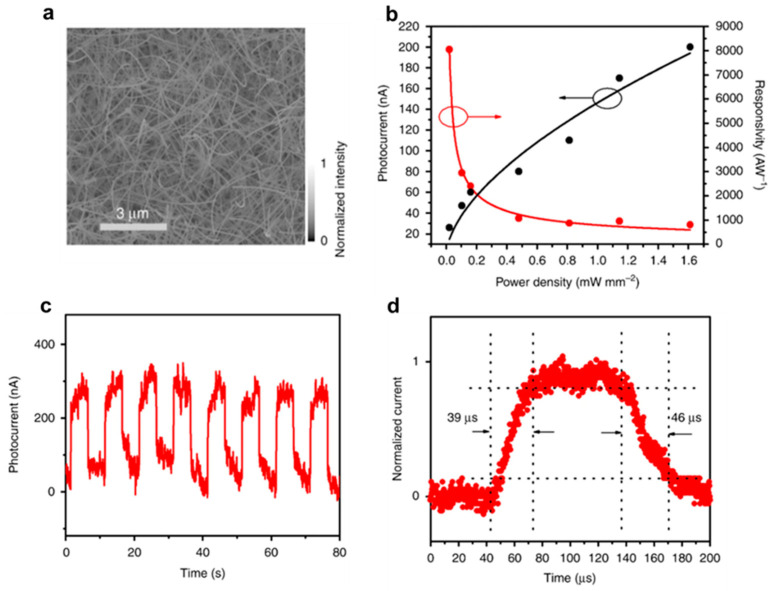
(**a**) Scanning electron microscopy image of In_0.28_Ga_0.72_Sb nanowires (NWs) prepared by using the precursor powder mixing ratio of InSb:GaSb = 40:1 (wt.) and 0.1 nm thick Au films as the catalyst; (**b**) Photocurrent (red, left y-axis) and responsivity (black, right y-axis) as a function of the incident illumination intensity; arrows indicate the mapping to the corresponding axes. (**c**) Photo response of the nanowire photodetector under the illumination intensity of 1.6 mW mm^−2^ with fixed frequency of 0.1 Hz; (**d**) A high-resolution transient photo response of the device to illustrate the rise time and decay time constants [48].

Given these outstanding features, the combination of their broad photo response range, superior electrical performance, robust stability, and the ability to easily form large-scale arrays makes them exciting prospects for next-generation optoelectronic devices and neuromorphic systems. Previous study, Li et al. [48] developed a photo detector utilizing In_x_Ga_1-x_Sb NW taking advantage of their high hole mobility and their ability to deliver broadband, ultrafast photo response across both the visible and infrared regions of optical communication (Figure 6). Figure 6a indicates longer than 10 μm of NWs are grown smooth and clean on the amorphous SiO_2_/Si substrate. And the curve of photocurrent and photoresponsivity as a function of the light intensity is demonstrated in Figure 6b. The relation between photocurrent and light intensity seems to be sublinear, opposite to photoresponsivity, which is easily observed in NW-based photodetectors. It is contributed by intricate mechanisms of electron-hole production, trapping, and recombination in NW. Figure 6c,d demonstrates the response speed and stability of the photodetector, which illuminated light with fixed frequency and intensity (0.5 Hz, 1.6 mW/mm^2^). The device shows effective switching properties, indicating remarkable periodicity and stability. Also, Figure 6d is calculated from high-resolution photo response data, and it shows rise and decay time to be 39 μs and 46 μs, respectively, demonstrating rapid response time due to the high hole mobility of NWs. Other recent studies including this research highlights the potential of 1D materials in optoelectronic devices. Through several findings including this research, 1D materials open up exciting possibilities for advancing optoelectronic devices and neuromorphic systems.

Beyond III-V semiconductor nanowires, a wide range of 1D metal oxide nanowires, including ZnO and IGZO, have recently drawn more attention for neuromorphic optoelectronic applications. Metal oxide nanowires are appropriate for large-area device integration because of their strong photo response, superior synaptic sensitivity, and chemical stability. For instance, Chen et al. [112] demonstrated that InZnO nanowire-based electrolyte-gated synaptic transistors could achieve improved synaptic sensitivity and long-term memory retention by precisely controlling oxygen vacancy concentrations through Ar plasma treatment. Similarly, Xin et al. [113] reported a heterojunctioni network composed of InZnSnO nanowires and perovskite quantum dots that exhibited negataive photoconductivity and highly efficient synaptic responses, enabling a visual recognition accuracy of up to 99% in artificial vision systems. Other studies have demonstrated the potential of SnO_2_ nanowire-based artificial synapses for bioinspired sensory systems. Yang et al. [114] integrated SnO_2_ nanowire synaptic devices into a neuromorphic gustatory system, achieving ultralow operating voltages (~1 mV), rapid response (<1 s), and long-term memory retention (>2 h) in taste perception and information processing tasks. These findings indicate that metal oxide nanowires are viable building blocks for next neuromorphic computing and optoelectronic devices due to their customizable electrical properties and suitability for large-area production.

### 3.3. Two-Dimensional Materials

2D materials such as graphene (Gr) [29,115,116], black phosphorus (BP) [117,118], and semiconducting transition metal dichalcogenides (TMDCs) [119,120,121] have emerged as key materials for optoelectronic devices due to their unique electrical, optical, and mechanical properties. These materials, being atomically thin, offer advantages such as flexibility, transparency, and excellent crystal quality, making them ideal for advanced optoelectronic applications [122,123]. Additionally, 2D materials can be fabricated through simpler and more cost-effective methods than traditional semiconductor materials, with integration into CMOS technology enhancing their scalability and performance.

**Figure 7:**
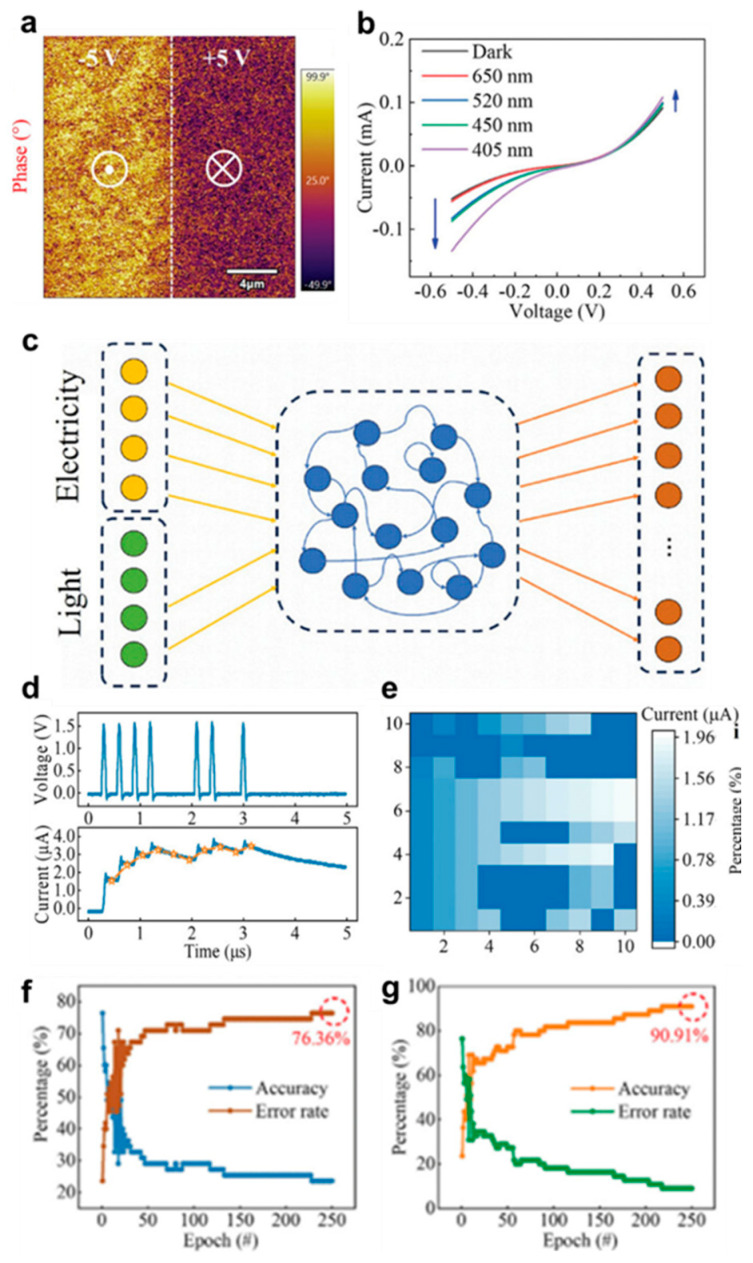
(**a**) PFM phase and amplitude images; (**b**) I–V characteristic curves of devices under the light with different wavelengths (650/520/450/405 nm). And the arrows indicates the I-V sweep direction; (**c**) Schematic diagrams of the structure of the computational network for mixed-input signals (optical and electrical signals) in reservoir pools; (**d**) The pulse waveforms and response current waveforms are applied corresponding to a single input signal and a mixed input signal. The orange curve shows one of the extracted results of the characteristic current characterization of the reservoir pools (1111001101), which is the sensing and processing of the 10-bit pulse train pairs; (**e**) The face image is converted into a pulse sequence and input into the perovskite memristor-based reserve pool system to read out the reserve pool status; (**f**,**g**) Recognition accuracy and error rates of reservoir computing networks under electrical/photoelectric signals for scaled images (#: Epoch number) [2].

The ability to tune their electronic properties through layer stacking and external stimuli has led to exciting advancements in bio-inspired optoelectronics, particularly in optoelectronic synaptic devices through several studies. Wang et al. [2] proposed a memristor based on 2D ferroelectric Ruddlesden-Popper (RP) perovskite film. Because of their structural diversity and tunability, 2D RP perovskites have ferroelectric functionalities in comparison to 3D. According to Figure 7a, domains of (BA)_2_(MA)_3_Pb_4_Br_13_ thin film shows obvious flip of polarization under different bias voltage, revealing the good ferroelectric polarization characteristics of perovskite ferroelectric films. Due to stable polarization and electron trapping, the device has respondence to light and it was found that as the light wavelength decreases, the I-V current rises, which is depicted in Figure 7b. Based on this responsivity, the device performed as memristor through RC. As the input is both optical and electrical signals, as Figure 7c, then the results of the current output exhibit as in Figure 7d,e. When a reservoir cell is capable of receiving both optical and electrical signals to perform optoelectronic mixed-input reservoir computation, the output response of the reservoir cell improves, leading to a higher recognition accuracy (Figure 7f,g). As this finding, several properties of 2D materials, such as their flexibility, transparency, and excellent crystal quality, combined with the ability to tune their electronic characteristics through layer stacking and external stimuli, make them highly promising for the development of advanced optoelectronic devices.

### 3.4. Fabrication Feasibility and Integration Challenges

Despite the outstanding optoelectronic and synaptic performance of hybrid material systems such as halide perovskites, QDs, and 2D heterostructures, significant obstacles remain in terms of fabrication and large-scale integration with CMOS technology. These materials frequently have inherent restrictions that render them unsuitable for traditional back-end-of-line (BEOL) semiconductor processes, which employ high-temperature thermal cycles and severe chemical treatments.

Wu et al. reported that a major obstacle to CMOS integration of perovskite-based devices is their chemical incompatibility with standard lithographic processes [124]. Perovskites are highly soluble in the polar solvents typically used as photoresist developers, and there is currently no universal wet or dry etchant capable of selectively and reproducibly etching perovskite films. These issues significantly complicate the patterning and scalability of perovskite devices in large manufacturing areas. Also, they are challenging to include in CMOS processes due to the characteristic that they typically demand heat budgets of 200–400 °C. Recent innovations, including low-temperature solution processing, nano-imprint lithography, and interface bonding techniques, have shown promise in addressing these challenges, but scalable and reproducible manufacture remains a substantial barrier [125].

Similarly, QD-based devices have issues with electrical instability and film homogeneity plague. Monolithic integration with CMOS circuitry is hampered by residual organic ligands and inadequate control over large-area deposition, which can result in performance variance and decreased yield [126]. In order to improve the stability and scalability of QD-based systems, sophisticated ligand engineering, strong surface passivation, and low-temperature deposition techniques will be needed. According to Yun et al., CuInSe_2_ PQDs can mitigate these limitations by replacing long organic ligands with inorganic halide ions, thereby enhancing film robustness and carrier mobility while enabling CMOS-compatible spin-coating of both p- and n-channel devices [127].

2D heterostructures such as graphene, MoS_2_, and h-BN present distinct problems. Although their atomic thinness and flexibility make them appealing, large-area uniform growth is still challenging to achieve [128,129]. Transfer-based stacking approaches can introduce interfacial defects and strain, leading to poor reproducibility. Hybrid integration of 2D devices with silicon-based neural circuits has been demonstrated at small scales, but scaling these processes to wafer-level remains a considerable hurdle.

Looking forward, advancements in a number of fields will be necessary to close the gap between CMOS-compatible neuromorphic systems and high-performance hybrid materials. Achieving dependable and scalable integration will also require co-design strategies that match the device structure, circuit specifications, and process limitations. Hybrid optoelectronic synaptic devices can become closer to feasible large-scale neuromorphic systems by resolving these fabrication and integration issues.

## 4. Optically Facilitated Synaptic Properties

It has been shown that humans obtain information from the external environment through vision [130]. The human retinal structure, one of the visual pathways, consists of several cells such as ganglion cells, bipolar cells, and photoreceptors [131]. In the retina, image information is carried by optical signals, which are converted into the changes in the neuron membrane potential and transmitted to the visual cortex [132]. To emulate this system, optoelectronic devices have been employed to create neuromorphic visual systems. Light has advantages with fast response, low energy consumption, and low cross talk [133,134]. Also, it enables precise synaptic weight modulation, mimicking retinal processes that are essential for learning and memory functions in neuromorphic systems [135,136,137].

### 4.1. EPSC

**Figure 8:**
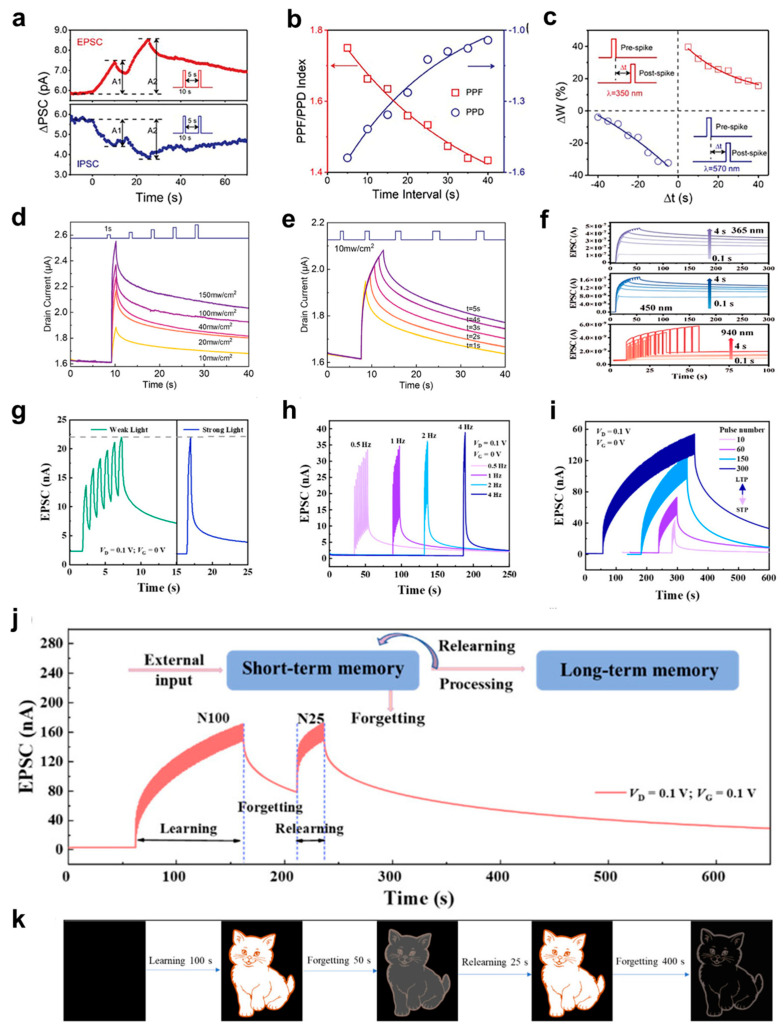
(**a**) The EPSC and IPSC in response to a pair of light pulses (interval of 5 s). Synaptic plasticity under light application; (**b**,**c**) Short-term and long-term PPF/PPD as the function of Δt [138]; The EPSC with (**d**) different light pulse intensities, (**e**) different light pulse width [49]; (**f**) The EPSC with different pulses width at various light wavelength [140]; (**g**) EPSC equivalent to that of a strong stimulus by accumulating weak stimuli; (**h**) Filter effect at different frequencies; (**i**) EPSC with different pulse numbers; (**j**) Learning-forgetting-relearning characteristics; (**k**) Simulated cat images to demonstrate distinct learning, forgetting and relearning behaviors [77].

The photocurrent reflects the synaptic plasticity of optoelectronic device, which is demonstrated by the ability of light activation to modify its resistance. Shan et al. [138] shows optoelectronic memristor that modulates synaptic plasticity all-optically to emulate the biological retina as shown in Figure 8a–c. A flexible plasmonic optoelectronic memristor was designed by investigating the nanocomposite film consisting of Ag-TiO_2_ hybrid nanoclusters and sodium alginate (SA) film. Due to the obvious absorption band of the Ag-TiO_2_ nanoclusters in the visible range between 400 and 750 nm, the experiments were conducted using UV light at 350 nm and visible light at 570 nm. To manifest short-term plasticity, related to the recognition and processing of data that can be easily forgotten [139], paired-pulse facilitation (*PPF*) and paired-pulse depression (*PPD*) were implemented by applying two UV and visible light pulses with pulse width 10 s and time interval varying from 5 s to 40 s, as introduced in Figure 8a,b. A_1_ and A_2_ refer to post synaptic current due to the first and second pulses. Also, *PPF* and *PPD* index varied by interval time is fitted with exponential equation:

(1) $$PPF\;or\;PPD\;index=Ae^{-∆t/\tau }+C$$

where A is the facilitation constant and τ refers to relaxation time (interval time). This shows shorter interval time enhanced *PPF*/*PPD* behaviors. Additionally, the temporal correlation caused by paired spikes can facilitate long-term potentiation (LTP). As shown in Figure 8c, the long-term synaptic weight change (∆W) resulting from *PPF* and *PPD* was measured under the paired UV and visible light spikes. ∆W is defined as $∆W=\;\left(G_{1}-G_{0}\right)/G_{0}$, where $G_{0}$ is the initial conductance and $G_{1}$ is the conductance measured in 10 s after the application of the paired spikes. Consequently, the long-term plasticity also rises as the interval time between spikes decreases. Yang et al. [49] researched the similar type of photo induced current response by using various light conditions in photoelectronic neuromorphic ferroelectric thin film transistors (FeTFTs) device. Photo induced current, also called EPSC was gained through applying light pulses under different light power intensity, light pulse width, and the number of light pulses, as depicted in Figure 8d–f. As the light pulse is applied, the channel current sharply increases and then gradually decreases once the light is turned off. Both the peak current and relaxation time (the duration it takes for the current to decay from its peak to the pre-stimulus level) grow as the light intensity increases, as demonstrated in Figure 8d. Similarly, Figure 8e shows that both current and relaxation time increases notably with longer pulse widths, where the light pulse intensity is fixed at 10 mW/cm^2^, with pulse widths ranging from 1 to 5 s. Moreover, Sui et al. [140] implemented experiments by applying ten pulses of different pulse widths at different wavelengths from 365 to 940 nm (6 mW/cm^2^, 0.5 s), which is illustrated in Figure 8f. This finding confirms that synaptic response by wavelength of 365 nm resulted in the largest EPSC, whereas the wavelength of 940 nm induces the smallest EPSC. As demonstrated in the above EPSC experiments, the modulation of synaptic plasticity through light activation highlights the potential of optoelectronic devices to mimic biological retinal processes, enhancing learning and memory functions in neuromorphic systems.

### 4.2. Visual Adaptation and Flicker Fusion

Visual adaptation in the human visual system refers to the process by which the eyes adjust to different light conditions [141]. In a dark environment with weak light, the pupil dilates to allow more light in, enabling the retina to accumulate and amplify faint light [142]. This ability allows the human eye to maintain vision across a wide range of lighting conditions. Huang et al. [77] (adapted from the same study as Figure 2) showcased the dextran-optoelectronic synaptic transistor (OST) that exhibits neuromorphic computation ability with synaptic plasticity. Figure 8g suggests the visual adaptation behavior of the dextran-OST. It reveals 6 consecutive low intensity (8 μW/cm^2^) light pulses reached comparable current level as it does when exposed to a single high intensity (30 μW/cm^2^) light pulse.

In addition, flicker fusion is a common visual adaptation mechanism that occurs when the frequency of light is raised to a particular point, human retina recognizes individual consecutive lights as continuous [143,144]. According to this theory, the human retina functions similarly to a low-pass filter, attenuating high-frequency signals and helping to prevent visual fatigue from such stimulus [145,146]. As Figure 8h supports, at low frequencies (0.5 Hz and 1 Hz), the EPSC increases gradually over time, showing clear flickering. However, as the frequency increases (2 Hz and 4 Hz), the response becomes more continuous and smoother, resembling a steady signal, similar to perception of human visual system when the flicker frequency exceeds the flicker fusion threshold. The ability to simulate visual adaptation and flicker fusion behavior demonstrates their potential for replicating key visual processing functions, opening doors for more advanced bio-inspired optoelectronic systems.

### 4.3. Transition from STP to LTP

Synaptic plasticity plays a crucial role in learning and memory. Short-term plasticity (STP) occurs when repeated stimuli leads to a temporary increase in synaptic strength [147]. If stimuli are sustained, this can transition into long-term plasticity (LTP), a more stable and lasting enhancement in synaptic strength [148]. This is fundamental for encoding new information and forming memories in biological systems. In neuromorphic systems, replicating and transitioning from STP to LTP is essential for building devices capable of learning, memory retention, and adaptation [149,150]. As shown in Figure 8i, increasing the pulse count (10–300) and pulse width (10–30 s) led to a rise in EPSC, with a longer recover time, indicating a shift from STP to LTP. The “learning-forgetting-relearning” behavior, a key indicator of learning and memory, is also evident. In Figure 8j, after learning with 100 light pulses, the EPSC peaked and gradually decreased. Following a 50 s forgetting period, the EPSC remained significantly higher than the initial value, showing strong memory retention. During a second learning phase with fewer pulses (25 pulses), the EPSC surpassed the first learning peak, indicating the OST’s ability for association and relearning. Additionally, after the second learning phase, the forgetting time increased to 400 s, demonstrating enhanced memory retention. This suggests that dextran-OST has great potential for deep learning applications. In Figure 8k, the normalized EPSC was mapped to the brightness changes in a “cat” image. Initially, the EPSC was normalized after 100 pulses (N100), and the corresponding image was recorded. After 50 s of forgetting (N100), followed by a relearning phase with 25 pulses (N25), the image sharpened. However, after 400 s of forgetting (N25), the image blurred again, demonstrating the dextran-OST’s ability to simulate memory processes like learning, forgetting, and relearning. This emphasized the importance of mimicking synaptic plasticity for the development of systems capable of learning, memory retention, and adaptation.

### 4.4. Nociceptor

Nociceptor, a sensory receptor responsible for detecting harmful or potentially damaging stimuli, is activated by noxious stimuli and transmits signals to the central nervous system [151]. A nociceptor determines noxious stimuli based on whether it exceeds threshold, and this principle applies to visual nociceptors as well, which respond to light [152]. Furthermore, visual nociceptors exhibit characteristics response such as “no adaptation”, “relaxation”, and “sensitization”, which occur under repetitive or stronger injury conditions [153,154]. The threshold, which spark nociceptor, is determined by several factors. In case of light, the duration, intensity, frequency, and the number of light pulses can be the activator.

**Figure 9:**
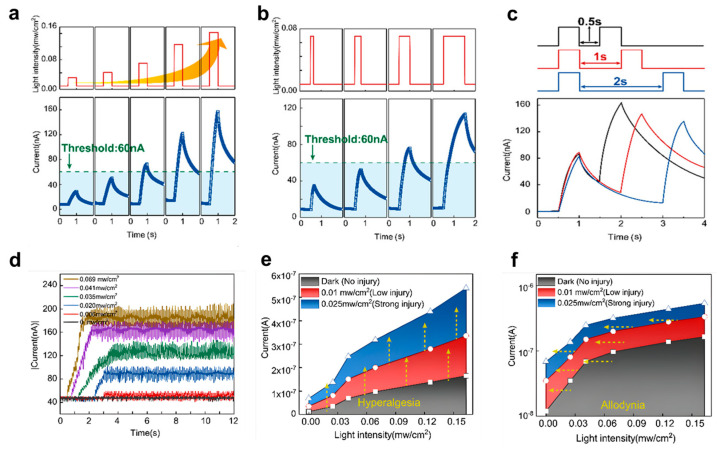
(**a**) Applying optical pulses with 0.5 s width and different pulse intensity from 0.025 to 0.163 mW/cm^2^; (**b**) A fixed optical stimulus (0.069 mW/cm^2^) with different pulse widths (0.15–1 s); (**c**) The resulting EPSCs using 0.069 mW/cm^2^ light pulse train followed by 0.069 mW /cm^2^ pulse train with different time intervals from 0.5 to 2 s; (**d**) The response of device to continuous multiple pulses with different light intensities (0, 0.006, 0.020, 0.035, 0.041, and 0.069 mW/cm^2^, respectively); The maximum output currents at different input voltage amplitudes (**e**) in linear scale and (**f**) in log scale, demonstrating the shift in the output current toward higher currents (Hyperalgesia) and light intensity that occurs threshold toward a lower threshold (Allodynia) [50].

Gong et al. [50] introduced three-terminal flexible memory phototransistor (MPT) that simulates the visual nociceptive behavior owing to the filled trapped states with electron-hole pair generated by the photocurrent. In Figure 9a,b, they defined the threshold current to 60 nA and observed the increases in current under differing light intensity and duration, respectively. As the pulse intensity reached 0.069 mW/cm^2^, the output current overcomes the threshold current (Figure 9a) with the fixed light pulse duration of 0.5 s. Also, under constant light intensity of 0.069 mW/cm^2^, at least 0.5 s is needed to surpass threshold (Figure 9b). Observation of threshold properties under various pulse intensity from 0 to 0.069 mW/cm^2^ is shown in Figure 9d. It reveals that under 0.006 mW/cm^2^, any considerable response is presented. And when exposed to continuous light pulses, it reaches saturation after the rise in current under the intensities greater than 0.02 mW/cm^2^. Additional pulses did not change the saturation current anymore, which indicates the balance between the generation and capture of light-generated electron-hole pairs and emulation of no adaptation characteristic of nociceptors. Next, relaxation is also one of the important nociceptive properties. By varying intervals between light pulses from 0.5 to 2 s, it appears that the device is more sensitive to stimuli when it is more relaxed (Figure 9c). Lastly, sensitization trait, which is a mechanism related to protecting injured areas by enhancing pain. It is categorized by two: hyperalgesia and allodynia. Hyperalgesia is a heightened pain in response to normal pain stimuli and allodynia refers to the pain caused by normally painless stimuli. Injured state was executed through illuminating high ultraviolet and normal stimuli is implemented by using low ultraviolet pulses. Linear and logarithmic scales are used to express the photocurrent as a function of light intensity as depicted in Figure 9e,f. It clarifies in order to activate a device that is injured, a lower threshold light intensity is needed which corresponds to allodynia and hyperalgesia. In summary, nociceptor emulation offers a promising approach for emulating sensory responses to harmful stimuli, contributing to the development of more sophisticated and responsive neuromorphic systems.

### 4.5. Associative Learning

Associative learning behavior is when an organism learns to link a specific stimulus with a response [155,156]. It allows them to anticipate outcomes and adjust behavior accordingly. Thus, this type of learning is crucial for survival as it enables organisms to predict and respond to changes in their surroundings. In the context of optoelectronic systems, associative learning can be observed when light stimuli are linked with specific responses, such as neural activity or synaptic changes.

**Figure 10:**
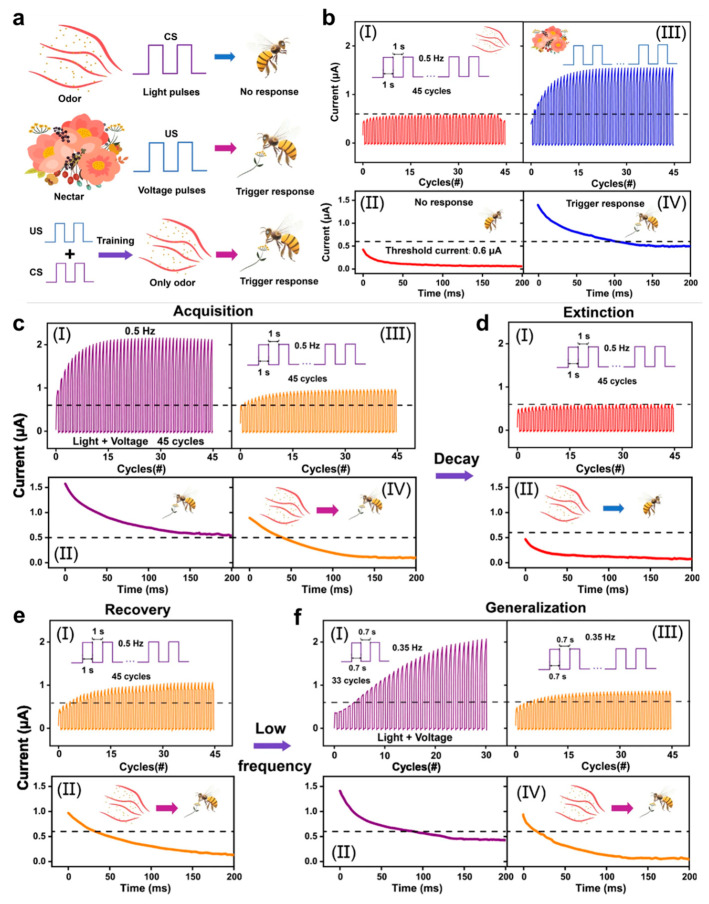
(**a**) Schematic of the classical PER of a honeybee. Light pulses emulate the CS (odor, no trigger response), and voltage pulses mimic the US (nectar, trigger response); (**b**) (I) Conductance response after excitement by CS (45 cycles of purely light pulses, 0.5 Hz) and (II) the decayed response within 200 ms after being excited by CS. (III) Conductance response after excitement by US (45 cycles of purely voltage pulses, 0.5 Hz) and (IV) the decayed response within 200 ms after being excited by US; Implement the four features in classical PER: (**c**) acquisition, (**d**) extinction, (**e**) recovery, and (**f**) generalization (#: pulse number) [158].

Wang et al. [157] proposed an optoelectronic memristor based on Ag/TiO_2_ Nanowires: ZnO quantum dots/FTO that is designed to emulate biological associative learning behaviors, specifically classical conditioning. The device mimics the four key features of classical conditioning— acquisition, extinction, recovery, and generalization—by associating light and voltage stimuli to trigger reflexes like responses, similar to how conditioned stimuli (CS) and unconditioned stimuli (US) interact in biological systems. Associative learning behavior was demonstrated using the proboscis extension response (PER) of honeybees as a model, as shown in Figure 10. To identify the presence of PER, a read pulse (0.1 V, 2 ms) was administered during the experiment, and the threshold current was set at 0.6 μA. The light pulses were designated as CS, which triggers unconditioned response (UR). And voltage pulses were designated as US, which induces conditioned response (CR) (Figure 10b). The four features mentioned above correlate to how the biological brain stores information, removes outdated information, remembers information, and stores new information. As demonstrated in Figure 10c, concurrent application of light pulse (CS, 0.5 Hz) and voltage stimuli (US, 0.5 Hz) induces rapid increase in conductance above the threshold, triggering PER. Through these associative pairs, PER was sustained until 200 ms. Following training, applying CS allows appearance of PER behavior, in contrast to Figure 10b due to training. After 10 min, light stimulus (CS, 0.5 Hz) was applied, and it failed to overcome threshold current (Figure 10d). However, the initial decay current upon training is slightly larger than that of the untrained instance (Figure 10b(II)), indicating insufficient neural link between the US and the CS. For recovery, identical associative pairs were applied to the device and activated rapidly with large magnitudes. Figure 10e implies that the neural link between the US and the CS has been re-strengthened, because the CR and decaying current is enhanced compared to previous acquisition. Lastly, in the generalization phase, low-frequency stimuli (light at 0.35 Hz and voltage at 0.35 Hz) were given after recovery, triggering the PER with a slower process. Although the response was weaker and decayed faster than high-frequency training, the CR still increased, confirming that the CS could trigger the PER. The generalization showed that while similar stimuli could trigger the response, they produced a lower CR with a faster decay. The demonstration of associative learning behaviors in optoelectronic systems provides a significant step toward creating devices that can mimic biological learning processes, enabling more intelligent and adaptable systems in neuromorphic computing.

### 4.6. Reliability and Long-Term Performance

Optoelectronic synaptic devices still face significant hurdles in providing long-term stability and endurance, in addition to achieving desired synaptic capabilities. This is because photo-generated carrier trapping/detrapping and oxygen vacancy migration are inherently reversible processes and typically exhibit poor retention [158]. Jeon et al. [159] also observed that ZnO nanoparticle-based optoelectronic devices exhibited rapid EPSC decay under UV light, highlighting the limited retention of photo-induced states.

Recent studies have made notable progress in addressing these reliability challenges. Under purely optical stimulation, for example, Zhu et al. [92] showed that a BP/CdS heterostructure-based artificial photonic synapse maintained robust long-term memory characteristics, with repeatable long-term potentiation (LTP) and long-term depression (LTD) switching beyond 150 cycles, and persistent postsynaptic current levels remaining stable for over 5000 s using low-power light pulses. Lee et al. [160] similarly reported that IGZO-based photonic neuromorphic devices, utilizing the PPC property of amorphous oxide semiconductors, can replicate both short-term and long-term memory functions under UV light. Frequency-dependent photonic stimuli were found to produce stable LTM retention for prolonged periods of time. The authors explained that during optical stimulation, long-lived ionized oxygen vacancies in IGZO maintain high conductance and allow for the establishment of LTM because they are unneutralized for prolonged periods of time.

In contrast to electrically programmed devices, long-term retention and environmental durability under simply optical stimulation are still limited despite these advancements. Over time, the photoactive layers and interfaces are frequently weakened by moisture intrusion, photooxidation, and thermal stress, which causes performance drift and variability. Future studies must concentrate on hybrid optoelectronic modulation approaches, sophisticated encapsulation techniques, and defect engineering to get around these restrictions and prolong device lifetime while guaranteeing reliable performance under real-world circumstances.

## 5. Applications

### 5.1. Potentiation and Depression for Artifitial Neural Network

**Figure 11:**
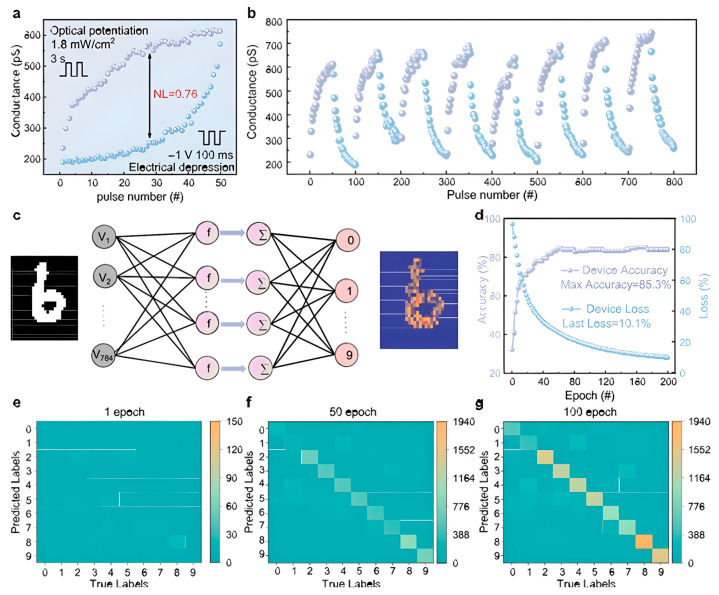
Image recognition utilizing a photoelectric memristor. (**a**) The nonlinearity of weight update (NL); (**b**) Stability and repeatability of LTP/LTD in the photoelectric memristor over eight cycles (#: pulse number); (**c**) Schematic representation of ANN architecture; (**d**) Variation in recognition accuracy and loss rate as a function of training duration (#: epoch number); (**e**–**g**) Average confusion matrix obtained from over 100 training sessions [161].

Figure 11a presents the measured nonlinearity of 0.76, indicating its suitability for training in the Modified National Institute of Standards and Technology (MNIST) pattern recognition system [161]. The stability and reproducibility of the memristor’s synaptic behavior were further examined, as shown in Figure 11b, where the device successfully retained consistent LTP and LTD characteristics over eight repeated cycles. These results confirm the robustness of the memristor for neuromorphic computing applications. To validate its computational functionality, the memristor was integrated into an artificial neural network (ANN) for handwritten digit classification. The network architecture, depicted in Figure 11c, consists of an input layer (784 neurons), a hidden layer (28 neurons), and an output layer (10 neurons), corresponding to MNIST digits from 0 to 9. The training process was conducted using the MNIST dataset. The experimental results, illustrated in Figure 11d, demonstrate a progressive increase in classification accuracy, reaching a peak of 85.3% after 200 training epochs. Figure 11e–g illustrate the step-by-step progression of the training process, demonstrating how the inferred output values gradually converge with the target values. These results validate the learning capability of the photoelectric memristor-based synapses, highlighting their effectiveness in facilitating accurate image recognition within neuromorphic computing systems.

### 5.2. Reservoir Computing Using Data Encoding Processing

**Figure 12:**
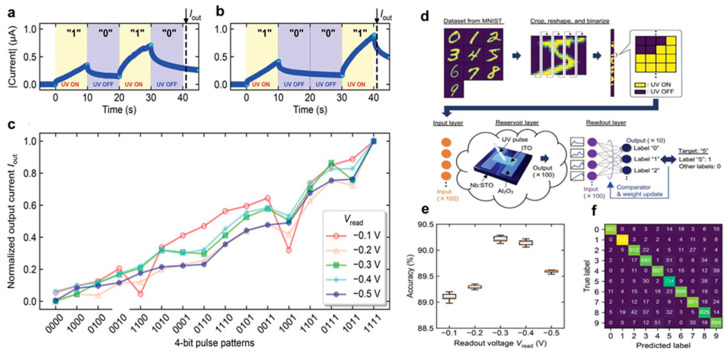
Device output response to a 4-bit encoded UV pulse at the ITO/Nb:STO junction. (**a**,**b**) Output currents corresponding to the 4-bit time-series inputs “1010” and “1001” at V_read_ = −0.5 V. I_out_ represents the current measured 1 s after the completion of the input sequence (indicated by the dashed line); (**c**) Normalized Iout values for 16 different 4-bit time-series input patterns, referenced to the “1111” input condition; Handwritten digit classification using a photonic memristor-based reservoir computing (PRC) system. (**d**) Schematic representation of the reservoir computing process implemented in this study; (**e**) Predicted classification accuracy for handwritten digits when utilizing the ITO/Nb:STO junction as a reservoir; (**f**) Confusion matrix for the MNIST dataset obtained at V_read_ = −0.3 V [163].

RC is an efficient framework for time-series signal analysis, ideal for edge computing applications [162]. It uses a “reservoir” to map input signals into a high-dimensional feature space, with the device’s nonlinear response serving as the physical reservoir. As depicted in Figure 12, an integrated multifunctional sensing and computing (IMSC) system was demonstrated using the ITO/Nb:STO device in an MNIST image classification task [163]. The device can function as a neuron or synapse, adapting to computational needs, and RC-based learning promotes parallel processing, self-organization, and adaptive connectivity.

The tunable time constant of the ITO/Nb:STO device makes it suitable for processing time-series signals. Figure 12a,b show the output currents for two 4-bit time-series inputs, “1010” and “1001,” measured under a V_read_ of −0.5 V. The time-series signals were encoded into the device using UV pulses, with the output current (I_out_) recorded 1 s after each 4-bit sequence. The output currents were 0.29 μA for “1010” and 0.65 μA for “1001,” indicating the device retains information about past inputs and effectively reflects the history of signal application. Figure 12c presents the output current for all 16 possible 4-bit input patterns. The Iout values were normalized relative to the response for the input sequence “1111,” showing how the output current varies based on the input sequence at different read voltages (V_read_). Notably, the output current increased as more “1”s appeared toward the end of the sequence, with each of the 16 input patterns producing a distinct Iout value, demonstrating the device’s ability to distinguish between different time-series inputs. For the pattern recognition (PR) task, the MNIST dataset was used. Figure 12d shows the schematic of the classification task, and Figure 12e illustrates the classification accuracy, with a peak of 90.2% achieved at a V_read_ of −0.3 V. This optimal performance was attributed to the effective alignment between the timescale of the input signals and the device’s relaxation time constant, suggesting that selecting a V_read_ corresponding to an appropriate current relaxation time can enhance learning efficiency. Figure 12f presents the confusion matrix for the MNIST dataset at V_read_ = −0.3 V, where common misclassifications involved digits “5” and “8” being identified as “3,” and “4” or “7” being misclassified as “9”. These specific misclassifications are also challenging for humans at a 20 × 20 pixel resolution. To clarify the specific contribution of the PR mechanism, the accuracy was evaluated by directly feeding preprocessed data (100 × 4 pixels) into the readout layer, bypassing the dynamic reservoir. This approach resulted in an accuracy of 85.1%, confirming that the ITO/Nb:STO junction improves classification accuracy while minimizing computational costs. Interestingly, when all 400 × 1 pixels were fed into a perceptron with 400 input nodes and 10 output nodes, the accuracy increased to 91.6%. This higher accuracy was due to the larger network size, demonstrating the trade-off between computational efficiency and network performance in the PRC system.

### 5.3. Adaptive LIF Neuron

**Figure 13:**
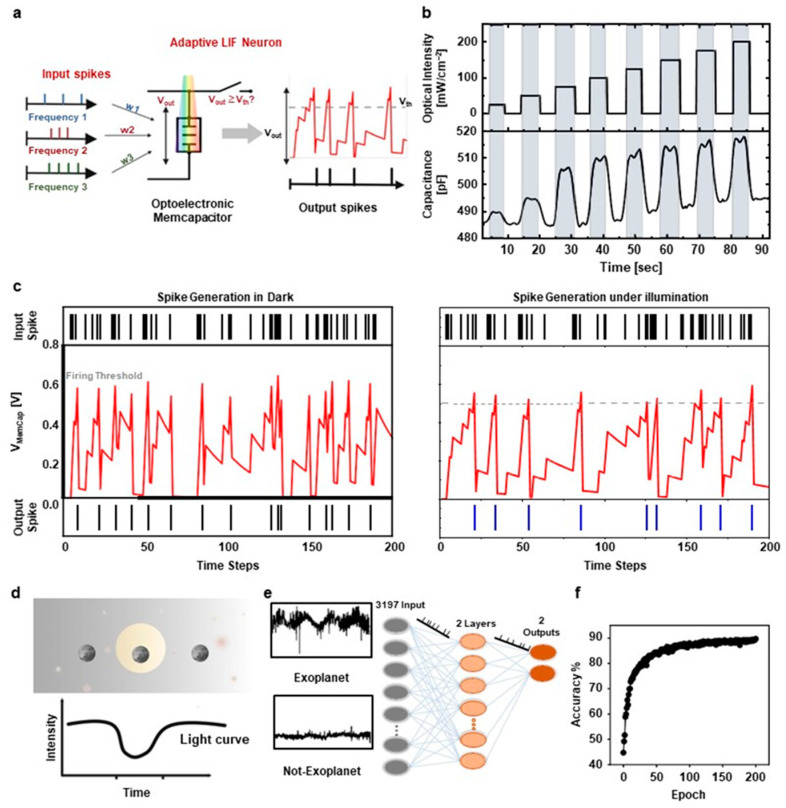
Adaptive LIF Neuron and exoplanet detection. (**a**) The LIF neuron generates spike frequencies based on varying input spike patterns from other neurons. The memcapacitor, which can be electrically and optically programmed, enables dynamic modulation of the neuron’s response characteristics; (**b**) CT measurements under varying light intensities (465 nm) show that the memcapacitor achieves eight discrete capacitance levels; (**c**) Simulation results confirm the adaptive behavior of the light-modulated memcapacitor. Higher capacitance values under 465 nm illumination (54.1 mW/cm^2^) lead to fewer output spikes by slowing down the charging and discharging process; (**d**) The transit method detects periodic brightness fluctuations in a star, indicating an exoplanet passing in front of it; (**e**) Illustration of a dynamic illumination-sensing neuron within an SNN framework for exoplanet detection, where each neuron processes temporal variations in the star’s light intensity; (**f**) Model testing accuracy improves over training epochs, reaching 90% [51].

Figure 13 show the behavior of the Leaky Integrate-and-Fire (LIF) neuron, which receives spike inputs from other neurons with different frequencies and generates an output spike when the integrated voltage exceeds a certain threshold [51]. The neuron, equipped with a memcapacitor that can be electrically and optically programmed, is capable of adaptive changes in its response properties. As illustrated in Figure 13a, this mechanism enables adaptive behavior in the neuron, allowing its spike frequency to dynamically adjust over time in response to different light stimulus patterns. As shown in Figure 13b, the memcapacitor exhibits notable variations in capacitance depending on the intensity of the incident light, reaching eight discrete levels with a minimum capacitance change of ΔCMemCap = 2 pF. The extracted capacitance values were applied in simulations based on Lapicque’s RC neuron model. As illustrated in Figure 13c, the simulation confirmed the neuron’s adaptive behavior under varying light conditions, where the spike frequency adjusted dynamically in response to changes in light intensity, exhibiting a distinct contrast to its behavior in the absence of light. In neuromorphic computing architectures, adaptive neurons are essential components in large-scale systems designed for high-efficiency computation. They contribute significantly to tasks such as pattern recognition, associative memory, and autonomous learning. Notably, adaptive neurons can be employed in Spiking Neural Networks (SNNs) for exoplanet detection utilizing the transit method, which is based on analyzing fluctuations in light intensity. This approach combines neuroscience, astronomy, and semiconductor technologies. As depicted in Figure 13d, this approach integrates concepts from neuroscience, astronomy, and semiconductor technology. The transit method identifies exoplanets by detecting periodic reductions in a star’s brightness when a planet crosses in front of it, partially obstructing its light. Traditional techniques for processing transit data often depend on sophisticated algorithms and extensive computational resources. In contrast, employing SNNs with adaptive neurons enables efficient and precise exoplanet detection while significantly reducing computational complexity. The proposed approach uses adaptive neuron models based on biological neuron behavior, enabling the neurons to adjust their sensitivity and response characteristics according to incoming signals. This adaptation allows the SNN to detect light intensity patterns associated with exoplanetary transits effectively. Figure 13e shows that the SNN architecture for exoplanet detection consists of layers of adaptive neurons connected through synapses. Each neuron receives input signals corresponding to temporal variations in light intensity from a target star. When light curve data is input into the network, neurons dynamically adjust their firing rates and synaptic strengths in response to the detected patterns. Adaptive neurons are employed in SNNs for exoplanet detection because they can operate in real-time and adapt to changing input conditions, achieving high accuracy (~90%) after 200 epochs of training (Figure 13f).

### 5.4. Colored Image Recognition

**Figure 14:**
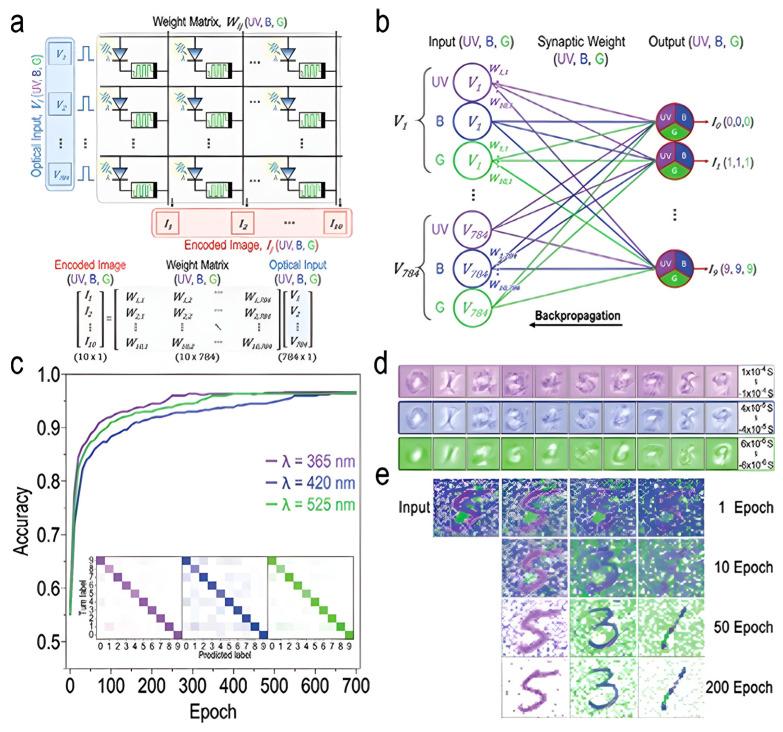
Colored and color-mixed image recognition by using the CsPbBr_3_ QD-based ONS device. (**a**) Schematic depiction of the multiply accumulate operation within a neuromorphic encoding process using a 2D 28 × 28 array of ONS devices. The bottom panel illustrates matrix-vector multiplication involving the input optical signal V_i_ (UV, B, G), synaptic weight W_ij_ (UV, B, G), and output current signal I_j_ (UV, B, G); (**b**) A single-layer ANN demonstrating the computational process for colored image recognition using ONS devices; (**c**) Recognition accuracy of UV, blue, and green-colored MNIST handwritten digits as a function of training epochs. Insets display confusion matrices of classification results after 700 training epochs; (**d**) Reconstructed synaptic weight mapping for MNIST patterns corresponding to ultraviolet (top), blue (middle), and green (bottom) color channels after 700 training epochs; (**e**) Encoded image (upper left) containing UV, blue, and green MNIST digits (5 in UV, 3 in blue, and 1 in green) mixed with background noise. Classification results obtained at training epochs of 1, 10, 50, and 200 demonstrate the network’s ability to distinguish MNIST digits across different color channels [52].

In order to implement neuromorphic vision systems, a study was recently conducted using a crossbar architecture and a tomographic artificial neural network with parameters extracted from ONS devices. Image data is encoded on optical neuromorphic synaptic (ONS) devices with 28 × 28 matrices, enabling both storage and computation for color image recognition [52]. Figure 14a presents the neuromorphic encoding process, where optical input signals in UV, blue, and green wavelengths are introduced to a 2D ONS device array. These inputs influence the synaptic weights, W_ij_ (UV,B,G), corresponding to each color channel. The encoded information is then processed through matrix-vector multiplication, adhering to Kirchhoff’s and Ohm’s laws. Figure 14b illustrates a single-layer neural network used for recognizing colored images. The input neurons represent the UV, blue, and green color channels, and they connect to the output neurons through synaptic weights that are adjusted according to color information. During the training process, these weights are optimized using the backpropagation algorithm to improve the accuracy of recognition. Figure 14c shows the training process using 60,000 MNIST datasets with different UV/B/G thresholds. The accuracy increases rapidly during training, surpassing 90% after the 200 epoch and leveling off. The confusion matrix confirms accurate recognition for digits across different colors. Figure 14d presents the synaptic weight maps for UV, blue, and green MNIST patterns after 700 training epochs, showing effective weight optimization for each color channel. Figure 14e displays an encoded MNIST image with UV/B/G digits and noise. The trained neural network successfully classifies the digits based on their color features, demonstrating the ability to recognize and differentiate colors, mimicking human visual processing. This work shows the potential of ONS devices for neuromorphic vision systems, enabling efficient and accurate colored image recognition.

### 5.5. Emerging Application Perspectives

In addition to the aforementioned applications, several studies highlight the broader potential of optoelectronic synaptic devices in next-generation neuromorphic architectures. For practical neuromorphic system integration, Li et al. [164] reported a wafer-scale PZT optical memristors fabricated via CMOS compatible sol–gel processes. These devices combine ultrafast volatile modulation based on the Pockels effect (48 Gbps, 432 fJ/bit) with stable non-volatile switching endurance exceeding 100,000 cycles, thereby enabling scalable and energy-efficient photonic-neuromorphic hardware platforms. Moreover, in the context of mechanical adaptability, Zhu et al. [165] conducted a comprehensive analysis of flexible memristors for neuromorphic computing, evaluating performance under bending, twisting, and stretching. The devices maintained stable resistive switching and synaptic functions even after repeated deformation, highlighting their potential for integration into wearable neuromorphic platforms and soft robotics. Extending beyond single-modality processing, Ji et al. [42] developed a memristive platform capable of simultaneously handling visual, auditory, and tactile inputs by encoding each modality into distinct conductance patterns. This multimodal integration mimics biological sensory processing and paves the way for adaptive artificial sensory systems. Collectively, these developments illustrate that the practical deployment of optoelectronic synaptic devices will necessitate a unified approach of system integration, mechanical flexibility, uniformity, and multimodal capability. Continued progress in these areas is expected to accelerate the transition from evidence of experiments to fully working neuromorphic systems for practical uses.

## 6. Conclusions

Optoelectronic synaptic devices represent a transformative approach to overcoming the limitations of conventional electronic computing. By leveraging light–matter interactions, these devices enable efficient synaptic plasticity, mimicking the adaptive nature of biological synapses. In this paper, we explored the fundamental mechanisms that govern optoelectronic synaptic behavior, highlighting key processes such as oxygen vacancy dynamics, defect trapping, and heterojunction-assisted charge modulation. Additionally, we examined how material dimensionality influences device performance, with 0D, 1D, and 2D materials offering unique advantages in charge transport, optical response, and integration capabilities. The discussion of synaptic properties demonstrated how optoelectronic devices can replicate essential cognitive functions, including excitatory post-synaptic photocurrent, visual adaptation, and associative learning. These features enable applications in artificial vision, reservoir computing, and neuromorphic hardware for complex data processing tasks. The integration of optoelectronic synapses in computing architectures has the potential to enhance energy efficiency and computational speed, paving the way for neuromorphic systems that outperform traditional semiconductor-based technologies.

Looking ahead, future research should focus on developing scalable, dependable, and system-level solutions rather than just proof-of-concept demonstrations. To achieve these goals, the development of semiconductors and heterostructures with inherent defect tolerance and environmental stability, defect-healing techniques, and multifunctional encapsulating layers will be necessary. Efforts should concentrate on enhancing energy efficiency by minimizing optical power demands via robust light–matter coupling structure (such as resonant cavities or plasmonic antennas) and low-energy programming methodologies, facilitating implementation in energy-limited edge–AI systems. In addition, it will be crucial to accomplish device uniformity and large-scale integration, necessitating the use of methodologies to reduce variability in conductance states across large crossbar arrays and wafer-level manufacturing approaches compatible with BEOL CMOS processes. Beyond device-level performance, research should broaden the application of optoelectronic synapses beyond conventional neural networks into advanced computational paradigms, including spike-based event-driven architectures, hybrid analog-digital computing, and real-time multi-model learning platforms. Validating these devices at the system level will also be crucial, requiring them to demonstrate intricate learning tasks in vast networks and compare them to state-of-the-art electronic and photonic neuromorphic systems using established assessment metrics. Lastly, stronger interdisciplinary collaboration among materials science, photonics, machine learning, and hardware system design will be necessary to accelerate the transition from lab-scale prototypes to deployable optoelectronic neuromorphic architectures.

## Figures and Tables

**Table 3:** Materials-based comparison of low-dimensional optoelectronic synaptic devices.

Material	Device	λ (nm)	Optical Intensity (mW·cm^−2^)	EPSC Magnitude	LTP Characteristics	Energy Consumption	Ref.
0D	P3HT–CsPbBr_3_ QD CNF	450	0.55–11.5	NR	>5000 s	0.18 fJ @ V_DS = −0.001 V, 50 ms	[84]
0D	CIGS/ZnSe QDs (PEC synapse)	450 (or 570)	0.3	NR	decay time~10^2^–6 × 10^2^ s	NR	[85]
1D	Single GaN nanowire	365	6.11 (range 0.21–6.59)	NR	NR	2.72 pJ (1 s, 5 V condition)	[86]
1D	ZnO nanowire device	365	0.099–0.134	NR	NR	~1 pJ (light 99 μW·cm^−2^, 1 s)	[87]
1D	SWCNT/ZnTPP phototransistor (channel: SWCNT; absorber: ZnTPP)	395	0.8–1	0.33 nA (200 ms, V_DS = 1 × 10^−7^ V)†	≥2 × 10^4^ s	6.5 aJ (200 ms, V_DS = 10^−7^ V)	[88]
2D	WSe_2_ (Lewis-acid doped)	532	0.6	NR	>1000 s	0.1 fJ (min)	[89]
2D	MoS_2_/Ta_2_NiS_5_ heterojunction transistor	532	NR	117.47 nA (200 ms)	~263 s	17.2 fJ (V = 1 mV)	[90]
2D	Bi_2_Se_3_ OES (2D thin film)	532	30.2	14.71 nA @ 2 V (532 nm)	~523.1 s	9.2 fJ (200 ms, 0.01 V)	[91]
2D	BP/CdS vdW photosynapse (channel: BP; photogate: CdS)	450	0.00016	~20 μA (ΔI at that intensity)	NR	4.78 fJ	[92]

## Data Availability

The original contributions presented in the study are included in the article, further inquiries can be directed to the corresponding author.
